# Alginate Oligosaccharides Prevent Dextran-Sulfate-Sodium-Induced Ulcerative Colitis via Enhancing Intestinal Barrier Function and Modulating Gut Microbiota

**DOI:** 10.3390/foods12010220

**Published:** 2023-01-03

**Authors:** Axue Wu, Yuan Gao, Ruotong Kan, Pengfei Ren, Changhu Xue, Biao Kong, Qingjuan Tang

**Affiliations:** 1 College of Food Science and Engineering, Ocean University of China, Qingdao 266100, China; 2 Qingdao National Laboratory for Marine Science and Technology, Qingdao 266100, China; 3 Department of Chemistry, Shanghai Key Lab of Molecular Catalysis and Innovative Materials, Fudan University, Shanghai 200438, China

**Keywords:** alginate oligosaccharides, guluronate oligosaccharides, mannuronic oligosaccharides, intestinal barrier function, gut microbiota, ulcerative colitis

## Abstract

Alginate oligosaccharides are degradation products of alginate and have attracted increasing attention due to their versatile biological functions. In the present study, C57BL/6 mice were used to assess the ameliorative effects and mechanisms of guluronate oligosaccharides (GAOS), mannuronic oligosaccharides (MAOS), and heterozygous alginate oligosaccharides (HAOS), which are the three alginate oligosaccharides of dextran sulfate sodium (DSS)-induced ulcerative colitis. The study showed that alginate oligosaccharides alleviated pathological histological damage by slowing down weight loss, inhibiting colonic length shortening, and reducing disease activity index (DAI) and histopathological scores. Alginate oligosaccharides modulated the colonic inflammatory response by reducing colonic MPO levels and downregulating the expression of IL-6 and IL-1β. Alginate oligosaccharides reduced intestinal permeability and reversed intestinal barrier damage by increasing the number of goblet cells, decreasing LPS levels, downregulating Bax protein levels, upregulating Bcl-2 protein levels, and enhancing the expression of the E-cadherin. Furthermore, alginate oligosaccharides modulated the composition of the gut microbiota and restored the production of short-chain fatty acids (SCFAs), especially acetate and butyrate. In conclusion, our study provides a scientific basis for the role of alginate oligosaccharides in relieving ulcerative colitis.

## 1. Introduction

Inflammatory bowel disease (IBD) consists of ulcerative colitis (UC) and Crohn’s disease (CD), for which the main characteristic is inflammation of the intestinal mucosa [1]. UC is a diffuse inflammatory response characterized by the presence of abscesses in the crypt, neutrophils, and plasma cells that infiltrate and attack the lining of the colon. Although the incidence of IBD has stabilized in Western countries [2], the prevalence of IBD is increasing yearly in developing countries where socioeconomic conditions are improving [3]. Studies have shown that DSS could cause symptoms of UC by inhibiting epithelial cell proliferation, disrupting the intestinal mucosal barrier and intestinal flora [4]. The experimental animals had symptoms of diarrhea, blood in the stool, and weight loss, accompanied by shortening of the colon and barrier dysfunction and inflammatory infiltration, which are most similar to the clinical symptoms and pathological changes of human UC [5].

The development of colitis has been shown to have an important association with the disruption of the intestinal barrier [6]. In the early stages of UC, the intestinal epithelial structure is disrupted and pathogenic micro-organisms in the intestinal lumen attack the intestinal epithelial cells. In addition, the expression of apoptosis-related proteins and anti-apoptotic proteins can be induced and inhibited by inflammatory factors in epithelial cells, respectively, which disrupts the tight junctions of epithelial cells, leading to intercellular gaps and affecting permeability [7,8]. Furthermore, the microbiota is also important for UC. Disruption of the gut microbiota structure finally leads to changes in the functions associated with the intestinal flora, which in turn aggravates the development of UC [9]. It was found that the intestinal flora of UC patients decreased in diversity and changed in composition; for example, the abundance of Firmicutes decreased significantly, while that of Bacteroidetes increased [10]. In addition, for microbial metabolites, it was found that SCFAs provide energy to colon cells and have anti-inflammatory, anti-tumor, and protective effects on intestinal barrier function [11]. Currently, the commonly used drugs for the treatment of ulcerative colitis include aminosalicylic acids (such as sulfasalazine, SASP), glucocorticoids and immunosuppressants, etc. However, pharmacotherapy has side effects such as indigestion and allergies. Therefore, there is an urgent need to find functional foods that have few side effects, are affordable, and have a significant intervention effect on UC.

Alginate, an acidic polysaccharide, is polymerized by α-L- guluronic acid (G) and β-D-mannuronic acid (M) in the form of 1,4 glycosidic bonds, and is mainly found in brown algae such as kelp, macroalgae, and sargassum [12,13]. Alginate oligosaccharides (AOS) are degradation products of alginate. There are three types of AOS according to the monomer sequences, which are as follows: guluronate oligosaccharides (GAOS) containing only G fragments, mannuronic oligosaccharides (MAOS) containing only M fragments, and heterozygous alginate oligosaccharide (HAOS) consisting of alternating M and G fragments [14,15]. Alginate oligosaccharides can exhibit a variety of biological activities such as immunomodulatory, antioxidant, and anti-inflammatory activities [16]. Previous studies have described that alginate oligosaccharides could reduce TNFR1-dependent apoptosis and thus maintain the integrity of intestinal epithelial cells [17]. Furthermore, it has been reported that AOS treatment could increase the abundance of beneficial gut microbes while inhibiting the growth of harmful bacteria in the intestine, and therefore maintain intestinal epithelial integrity [18].

Based on the above research, our study aimed to examine the effects of three conformations of alginate oligosaccharides, including GAOS, MAOS, and HAOS on DSS-induced ulcerative colitis and to explore their possible mechanisms.

## 2. Materials and Methods

### 2.1. Materials and Reagents

Three types of alginate oligosaccharides, namely guluronate oligosaccharides (GAOS), mannuronic oligosaccharides (MAOS), and heterozygous alginate oligosaccharides (HAOS), were provided by Qingdao BZ oligo Biotech Co., Ltd. (Qingdao, China). The relative molecular mass of GAOS is less than 8000 Da and the content is 83.1%. The relative molecular mass of MAOS is 6000 Da and the content is 82.5%. The molecular weight of HAOS is less than 4000 Da and the content is 92.5%. DSS (36–50 kDa) was supplied by Dalian Meilun Biotech Co., Ltd. (Dalian, China). Sulfasalazine (SASP) was supplied by Shanghai Yuanye Bio-Technology Co., Ltd. (Shanghai, China). The total DNA/RNA/Protein kit was obtained from Omega Bio-Tek (Guangzhou, China). The Omni-EasyTM One-Step PAGE Gel Fast Preparation kit and Omni-EasyTM Instant bicinchoninic acid (BCA) Protein Assay Kit were purchased from Shanghai Epizyme Biotechnology Co., Ltd. (Shanghai, China). The ELISA assay kits for IL-6, IL-1β, TNF-α, and MPO were supplied by Nanjing Jiancheng Biotechnology (Nanjing, China).

### 2.2. Animals and Experimental Design

Sixty male C57BL/6 mice (18–20 g, 6 weeks old) were purchased from Vital River Laboratory Animal Technology Co., Ltd. (Beijing, China). In the process of the experiment, the mice were exposed to 24 ± 1 °C, 55 ± 15% humidity, and a 12 h light and dark cycle, and were free to drink and eat.

**Figure 1:**
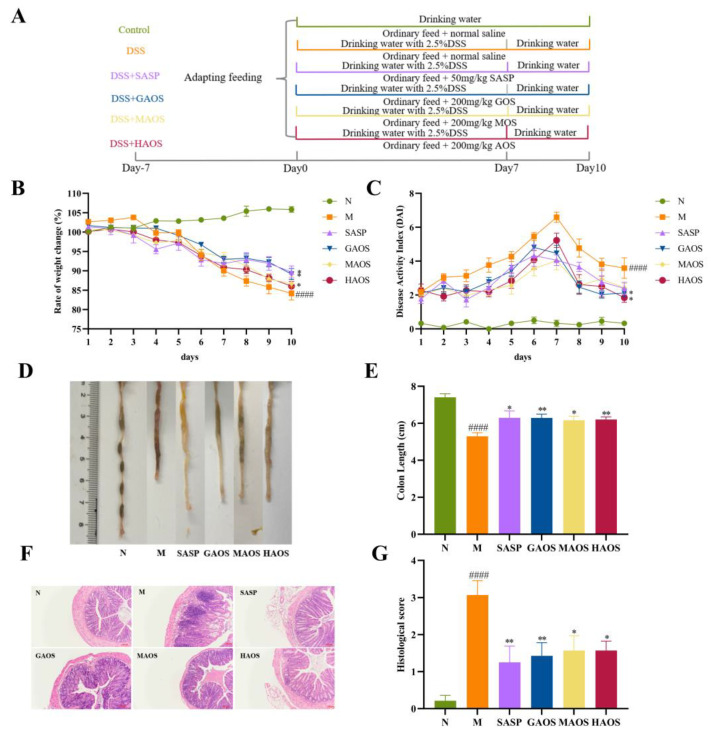
Effect of alginate oligosaccharides supplementation on histopathology of DSS-induced colitis. (**A**) Experimental design. (**B**) Rate of weight change (%). (**C**) Disease activity index (DAI). (**D**) Representative images of the colon from N, DSS, SASP, GAOS, MAOS, and HAOS groups. (**E**) Colon length of mice (cm). (**F**) Hematoxylin and eosin (HE) staining of colon tissues (magnification ×400). (**G**) Histological scores. Data were mean ± SEM (*n* = 8). #### *p* < 0.0001 vs. the N group; * *p* < 0.05, ** *p* < 0.01 vs. the M group.

After adaption for 7 days, mice were divided into the following 6 groups (*n* = 8): normal group (N), DSS-induced model group (M), DSS with Sulfasalazine (50 mg per kg body weight, SASP), DSS with guluronate oligosaccharides (200 mg per kg body weight, GAOS), DSS with mannuronic oligosaccharides (200 mg per kg body weight, MAOS), and DSS with heterozygous alginate oligosaccharide (200 mg per kg body weight, HAOS). The dosage of the three alginate oligosaccharides was selected based on previous studies [19]. The experimental design is shown in Figure 1A. The experimental period was from days 0 to 10, and the modeling period was from days 0 to 7. Mice in the normal group were fed and watered normally throughout the experimental period (days 0–10) and 200 μL saline was gavaged as the control. A total of 2.5 g of DSS was dissolved in 100 mL of distilled water to prepare a 2.5% solution of DSS; mice in the model group drank 2.5% DSS solution from day 0 to the end of the modeling period for 7 days and 200 μL saline was gavaged as the control throughout the experimental period, while the mice in the SASP, GAOS, MAOS, and HAOS groups were given 2.5% DSS solution from day 0 to the end of modeling for 7 days, and the corresponding doses of SASP, GAOS, MAOS, and HAOS solutions were gavaged throughout the experiment. Then, the mice were executed on day 11. The distal colon tissues were fixed in paraformaldehyde (4%) for histopathology. The serum, colon tissues, and intestinal contents of all mice were collected and stored at −80 °C refrigerators for further analysis.

The whole process of animal experiment was implemented based on the recommendations in the Guide for the Care and Use of Laboratory Animals of the National Institutes of Health and approved by the Committee on the Ethics of Animal Experiments of the Ocean University of China (Approved protocol no: SPXY2022022401).

### 2.3. Assessment of Colitis

During the entire experiment, the body weight of the mice was recorded daily to calculate the rate of weight change. Additionally, the disease activity index (DAI) score was calculated according to weight loss, fecal state, and occult blood condition (Table 1). After the mice were killed, the length between the ileocecum and proximal rectum was measured as the colon length and recorded.

### 2.4. Histological Analysis

About 1 cm of the distal colon was fixed with 4% paraformaldehyde, and then hematoxylin-eosin (H&E) staining, and alcian blue and periodic acid-Schiff (AB-PAS) were performed for histopathological analyses. The slices were observed by using a microscope under ×100 magnification (Olympus Optical Co., Ltd., Tokyo, Japan) and histopathological scoring was performed on HE sections (Table 2).

### 2.5. Biochemical Analysis

The blood of the mice was centrifuged at 4 °C and 5000× *g* for 15 min, and the upper layer of serum was used. Serum LPS content was determined in strict accordance with the instructions of the ELISA assay kit.

About 1 cm of colon was prepared in tissue homogenate with a 10% mass fraction using saline; it was then centrifuged at 4 °C and 5000× *g* for 15 min to obtain the supernatant. The contents of MPO, IL-6, IL-1β, and TNF-α in colon tissues were determined strictly according to the instructions of the ELISA assay kit.

### 2.6. Western Blotting Analysis

Proteins from the colon were extracted based on the instructions of the Total DNA/RNA/Protein kit. The concentration of the extracted protein was measured by using the BCA kit (Epizyme, Shanghai, China), and the protein concentration was diluted to 5 ug/uL. Afterward, a protein-loading buffer was used and boiled at 100 °C for 5 min to denature the protein. The general process of Western blotting can be summarized in the following steps [20]: the gel was prepared by using the Omni-EasyTM One-Step PAGE Gel Fast Preparation kit and equal amounts of proteins (30 ug) were injected into the gel. The prepared gel was placed in an electrophoresis tank with an electrophoresis buffer (Servicebio, Wuhan, China) which ran at 100 volts for 2 h. Next, the gel was transferred to the polyvinylidene fluoride (PVDF) membrane (Thermo Scientific, Waltham, MA, USA). The western blot transfer was done in wet conditions. Firstly, the gel and two filter papers were soaked in Tris-glycine transfer buffer (Servicebio, Wuhan, China) for one minute, while the PVDF membrane was activated in methanol for one minute. Then, the filter paper, gel, PVDF membrane, and filter paper were placed on the membrane transfer device from bottom to top, which is clamped tightly, with no air bubbles within it. It is important that the gel is closest to the negative electrode and PVDF membrane closest to the positive electrode. The condition of membrane transfer is 300 mA for 55 min. After the transfer, the membrane was blocked with 5% skimmed milk powder solution (Solarbio, Beijing, China) for two hours. Then, the PVDF membrane was cut according to the molecular weight of the measured protein and incubated with the primary antibody overnight. The next day, we continued to incubate the secondary antibody for two hours and then placed the trimmed PVDF membrane into the enhanced chemiluminescence tool (ABclonal, Wuhan, China) and performed scanning using an imaging system (Tanon, Shanghai, China).

Primary antibodies Bax (dilution 1:1000), E-cadherin (dilution 1:1000), and anti-rabbit secondary antibodies (dilution 1:3000) were purchased from Proteintech Group, Inc. (Wuhan, China). Primary antibodies Bcl-2 (dilution1:1000) and β-actin (dilution 1:10,000) were purchased from ABclonal Technology Co., Ltd. (Wuhan, China). All antibodies were diluted with 1 × TBST (Servicebio).

### 2.7. Short-Chain Fatty Acids (SCFAs) Detection

SCFAs of the cecum contents were determined by performing gas chromatography (GC) following the previously published approach, with some modifications [21]. Each 0.1 g thawed fecal sample was combined with 600 µL of ultrapure water and then homogenized. The homogenate was combined with 25 µL of 50% sulfuric acid, left to stand for 5 min, and stirred once per minute. Then, it was centrifuged at 5000× *g* for 10 min, and 300 µL of the supernatant was combined with 15 µL of 2-ethylbutyric acid and 150 µL of anhydrous ethyl ether. After thorough mixing, we performed centrifugation at 1000× *g* for 10 min and used 1 µL of the ether layer for subsequent analysis. A GC analysis was performed using an Agilent 6890 system, as previously described in [22].

### 2.8. 16S rRNA Gene Sequencing

The extracted DNA from feces in the colon of N, M, GAOS, and HAOS groups was measured according to QiaAmp DNA Stool Mini Kit (Qiagen, Hilden, Germany). The purity of the DNA was detected by Nanodrop 2000 (Thermo Fisher, Waltham, MA, USA) [23]. The V3–V4 region of the 16SrRNA gene was selected for amplification, as previously described [5].

### 2.9. Statistical Analysis

All data in this paper were presented as the mean ± standard error of the mean (SEM). The results were derived by using one-way ANOVA and *t*-test of GraphPad Prism 8 software. A *p* value < 0.05 was considered statistically significant.

## 3. Results

### 3.1. Alginate Oligosaccharides Treatment Alleviates the Histopathological Damage of Colitis Mice

From the third day of DSS administration, all groups except the N group showed a trend of weight loss. The tendency of mice to lose weight became slower after SASP and different alginate oligosaccharides treatment, and the SASP, GAOS, and MAOS groups significantly alleviated weight loss in contrast to the M group (*p* < 0.05), as shown in Figure 1B.

The DAI score and colon length represent the seriousness of colon injury. The DAI score in the DSS-induced M group was significantly increased in contrast to the N group, the maximum value was reached on the seventh day, and diarrhea and hematochezia were common in the M group (*p* < 0.0001). The DAI score of SASP, GAOS, MAOS, and HAOS treatment groups decreased, especially in the GAOS and HAOS groups (*p* < 0.05), as shown in Figure 1C. In addition, colonic hyperemia, edema, and colon length were significantly shortened in the M group (*p* < 0.0001), while colonic shortening was alleviated in the SASP and the three different structures of alginate oligosaccharides groups, as shown in Figure 1D,E.

Figure 1F showed that DSS-treated mice experienced severe damage to colonic epithelial cells, the disappearance of crypts, and the extensive infiltration of inflammatory cells. Additionally, the histological score of the M group mice was higher than that of the N mice (*p* < 0.0001). Colonic inflammatory infiltration, mucosal damage, and crypt loss were alleviated in SASP and different alginate oligosaccharides treatment groups. The histopathological score of the oligosaccharide groups with different structures significantly decreased, especially the SASP and GAOS groups (*p* < 0.01), as shown in Figure 1G.

### 3.2. Alginate Oligosaccharides Treatment Regulated the Inflammatory Response in Colitis Mice

**Figure 2:**
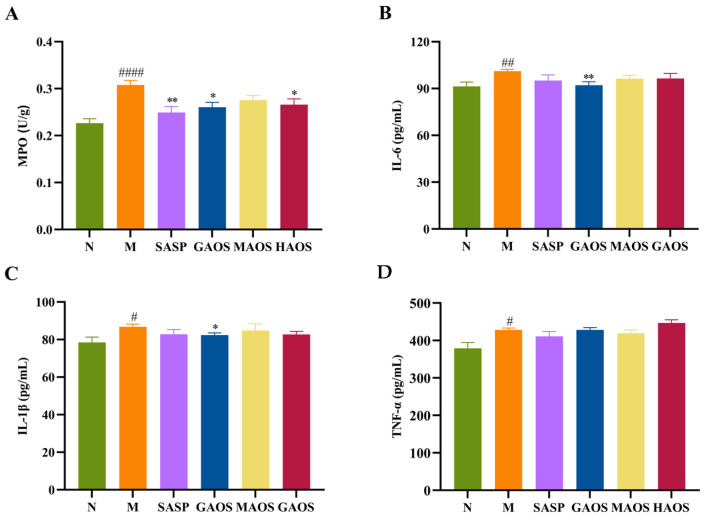
Effect of alginate oligosaccharides on inflammatory responses in mice with DSS-induced colitis. (**A**) MPO activity. (**B**) IL-6. (**C**) IL-1β. (**D**) TNF-α. Data were mean ± SEM (*n* = 8). # *p* < 0.05, ## *p* < 0.01, #### *p* < 0.0001 vs. the N group; * *p* < 0.05, ** *p* < 0.01 vs. the M group.

MPO levels in colonic tissues were measured to determine inflammatory cell infiltration. In our research, the activity of MPO (*p* <0.0001) in the M group significantly increased, as shown in Figure 2A. SASP (*p* < 0.01), GAOS (*p* < 0.05), and HAOS (*p* < 0.05) interventions significantly decreased the activity of MPO, and MAOS also showed a tendency to reduce MPO activity but not significantly.

Inflammatory cytokines are important in ulcerative colitis and their excessive production can exacerbate the inflammatory cascade, further damaging the colon [24]. We examined the levels of pro-inflammatory cytokines in colonic tissues. The expression of pro-inflammatory factors IL-6 (*p* < 0.01), IL-1β (*p* < 0.05), and TNF-α (*p* < 0.05) in the M group were significantly higher than those in the N group. Three types of alginate oligosaccharides downregulated the expression of IL-6 and IL-1β, with GAOS significantly reducing the levels of IL-6 and IL-1β, but the alginate oligosaccharides had no significant regulation effect on TNF-α, as shown in Figure 2B–D.

### 3.3. Alginate Oligosaccharides Treatment Regulates the Intestinal Barrier in Colitis Mice

**Figure 3:**
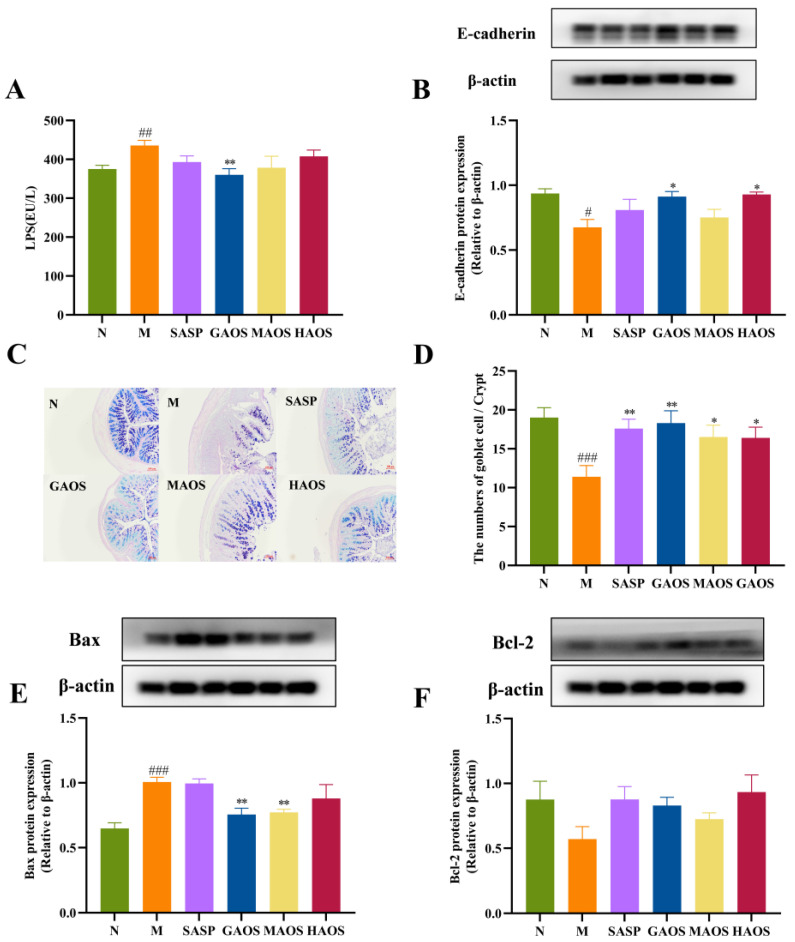
Effects of alginate oligosaccharides on the intestinal mucosal barrier in DSS-induced colitis mice. (**A**) The levels of LPS in serum (*n* = 8). (**B**) The protein levels of E-cadherin. The relative intensities of E-cadherin (*n* = 3). (**C**) Images of AB-PAS-stained sections of the colon (magnification ×400). (**D**) Goblet cell number per villus (*n* = 8). (**E**) The protein levels of Bax (*n* = 3). (**F**) The protein levels of Bcl-2 (*n* = 3). Data were mean ± SEM. # *p* < 0.05, ## *p* < 0.01, ### *p* < 0.001 vs. the N group; * *p* < 0.05, ** *p* < 0.01 vs. the M group.

The expression of LPS in the M group increased significantly (*p* < 0.01). In contrast to the M group, the level of LPS significantly decreased in the GAOS groups (*p* < 0.01), as shown in Figure 3A. Further, we measured the expression of E-cadherin in colonic tissues, as shown in Figure 3B. E-cadherin protein levels were significantly downregulated in the M group (*p* < 0.05) and significantly upregulated after GAOS and HAOS intervention (*p* < 0.05), suggesting that alginate oligosaccharides may be able to reduce intestinal permeability by increasing the expression of adherent junction proteins to reduce intestinal permeability.

The number of goblet cells was observed in colonic tissues through AB-PAS staining. In contrast to the N group, the number of goblet cells in the M group significantly decreased (*p* < 0.01), while the number of goblet cells was increased in SASP and alginate oligosaccharides with different structures groups, especially the SASP (*p* < 0.05) and GAOS (*p* < 0.01) groups, as shown in Figure 3C,D. On this basis, we further determined the expression of apoptotic proteins in colonic epithelial cells. As revealed in Figure 3E,F, the pro-apoptotic protein of Bax was significantly downregulated (*p* < 0.001); meanwhile, the anti-apoptotic protein of Bcl-2 was upregulated in the DSS-induced M group relative to the N group. The protein expression of Bax experienced an extremely significant decrease after GAOS and MAOS intervention (*p* < 0.01). Additionally, the protein expression level of Bcl-2 increased after the intervention of different conformations of alginate oligosaccharides, although the increase was not significant.

### 3.4. Alginate Oligosaccharides Treatment Promoted the Production of SCFAs in Colitis Mice

**Figure 4:**
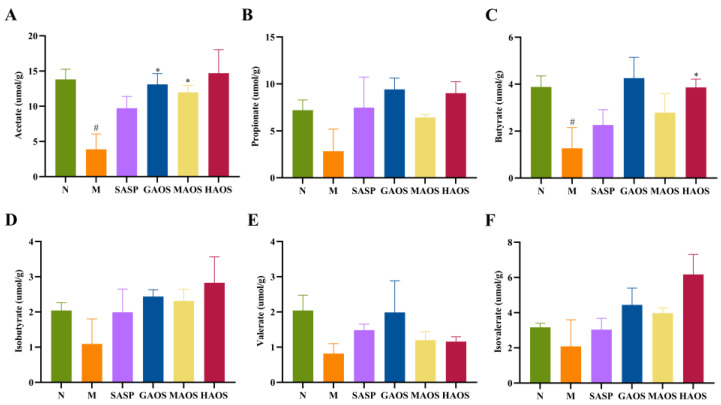
Effects of alginate oligosaccharides treatment on the SCFAs concentrations in DSS-induced mice. The concentration of (**A**) acetate, (**B**) propionate, (**C**) butyrate, (**D**) isobutyrate, (**E**) valerate, and (**F**) isovalerate in the cecal contents. Data were mean ± SEM (*n* = 8). # *p* < 0.05 vs. the N group; * *p* < 0.05 vs. the M group.

Alginate oligosaccharides cannot be absorbed in the digestive tract but directly transfer to the colon to interact with gut microbiota, which is used by intestinal microbes and degraded into SCFAs. Therefore, the contents of SCFAs in cecum were further analyzed and determined. The contents of acetate, propionate, butyrate, iso-butyrate, valerate, and isovalerate in the M group were decreased compared with those in the N group, especially acetate (*p* < 0.05) and butyrate (*p* < 0.05), as shown in Figure 4A–F. The production of SCFAs showed a significant trend of increasing after SASP and three different configurations of alginate oligosaccharides treatment. Notably, the GAOS and MAOS (*p* < 0.05) groups significantly improved the levels of acetate, and the HAOS (*p* < 0.05) group significantly improved the contents of butyrate compared with the DSS group.

### 3.5. Alginate Oligosaccharides Treatment Regulates the Gut Microbiota in Colitis Mice

**Figure 5:**
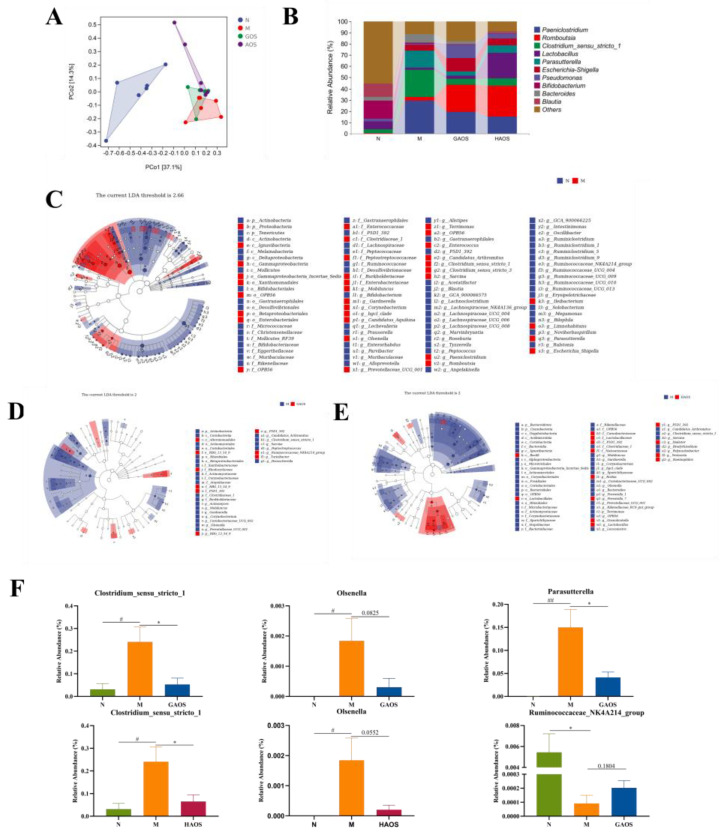
Effects of alginate oligosaccharides administration on gut microbiota in DSS-induced colitis mice. (**A**) Principal component analysis (PCA) with the cluster. (**B**) Microbial distributions of different groups at the genus level. LDA distribution. Linear discriminate analysis effect size (LEfSe) was performed to determine the difference in abundance; the threshold of the LDA score was 2.0. (**C**) The LEfSe analysis between N and M. (**D**) The LEfSe analysis between M and GAOS. (**E**) The LEfSe analysis between M and HAOS. (**F**) The relative abundance of *Clostridiaceae_1*, *Olsenella*, *Parasutterella,* and *Ruminoccaceae_NK4A214_group*. Data were mean ± SEM (*n* = 6). # *p* < 0.05, ## *p* < 0.01 vs. the N group; * *p* < 0.05 vs. the M group.

The dysregulation of the intestinal flora is thought to be closely related to UC, so GAOS and HAOS were selected for 16S rRNA sequences to investigate the effects of alginate oligosaccharides on intestinal microbes in colitis mice, which had more significant effects on colitis relief in this study. A principal coordinate analysis (PCoA) represents the compositional structure of the samples, and the samples with high similarity in the community structure are closer together. The β-diversity of intestinal microbes in the M group was significantly different from the N group, while GAOS and HAOS interventions prevent the bias of the gut microbiota, as shown in Figure 1A. Furthermore, Figure 5B showed the structure of the intestinal flora of mice treated with GAOS and HAOS at the genus level. In contrast to the N group, the gut microbiota structure of the DSS-induced M group was dominated by *Paeniclostridium*, *Clostridium_sensu_stricto_1*, and *Parasutterella*. *Romboutsia* and *Lactobacillus* increased after HAOS and GAOS treatment, which supported the result that GAOS and HAOS interventions changed the composition of intestinal flora.

LEfse facilitates a comparison between multiple groups and identifies species that differ significantly in abundance between groups. As shown in Figure 5F, both GAOS and HAOS downregulated *Clostridium_sensu_stricto_1* and *Olsenella* in contrast to the DSS-induced mice. Furthermore, GAOS downregulated *Parasutterella* and upregulated *ruminoccaaceae_nk4a214_group*.

### 3.6. Spearman Correlation between Colitis Indexes and Gut Microbiome

**Figure 6:**
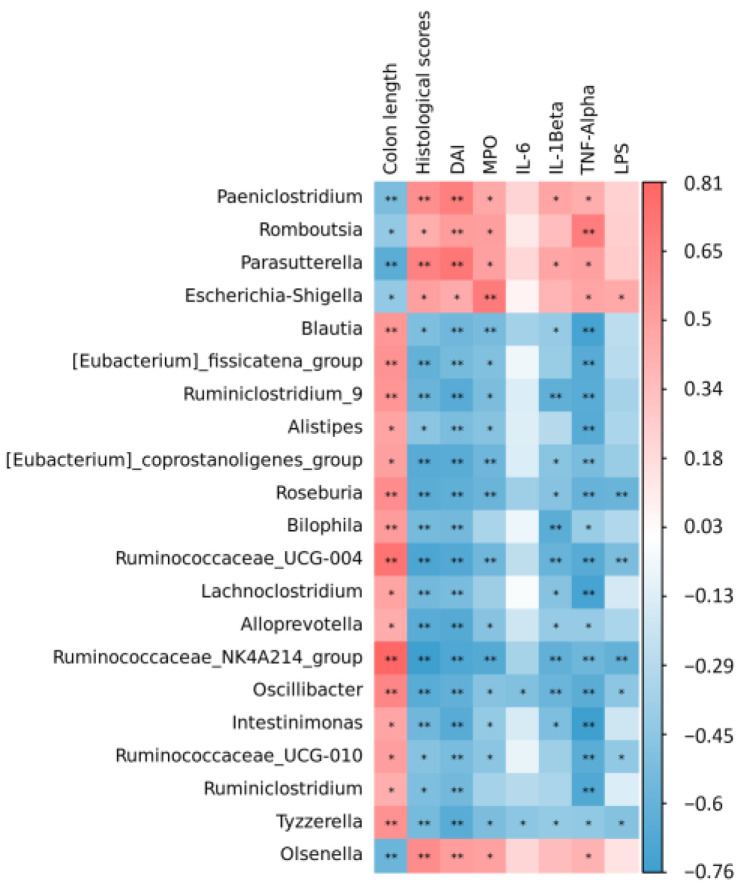
Spearman correlation between colitis indexes and top 50 genera in terms of abundance of the intestinal flora. Correlations with *p* < 0.05 were considered significant. * Significantly different (*p* < 0.05) between bacteria and colitis-related indicators by *t*-test, ** Significantly different (*p* < 0.01) between bacteria and colitis-related indicators by *t*-test.

To identify specific microbes that correlated with alginate oligosaccharides treatment in colitis mice, the Spearman correlation analysis was performed between the indicators associated with colitis and the top 50 genera of intestinal flora abundance in our research, and the results were shown in Figure 6. *Parasutterella* and *Olsenella* were significantly negatively correlated with colon length and positively correlated with the pathological histological score, DAI, MPO, and inflammatory factors. In addition, *ruminoccaaceae_nk4a214_group* presented a significant positive correlation with colon length and a significant negative correlation with the pathological histological score, DAI, MPO, LPS, IL-1β, and TNF-α.

## 4. Discussion

Previous research has established that dietary oligosaccharides play a vital role in modulating the gut microbiota, intestinal mucosal barrier, and inflammation. Alginate oligosaccharides were derived from the degradation of alginates and exhibited multiple bioactivities. This research aimed to illustrate the alleviating effect and mechanism of three types of alginate oligosaccharides (GAOS, MAOS, and HAOS) on DSS-induced ulcerative colitis.

The colon’s main function is to form, store, and excrete feces. If the function of the colon is disturbed, then symptoms such as diarrhea, constipation, and bloating will occur. Therefore, the DAI is often used to assess clinical status. Our findings indicated that three different configurations of alginate oligosaccharides effectively alleviate the symptoms of colitis, including weight loss, increased DAI, and a shortened colon. Histopathological examination is a clinically necessary method and tool to confirm the diagnosis of UC. Meanwhile, in the animal induction model of colitis, the pathological histological score is also used for evaluating whether the model has been successfully established. In our present study, the model group showed a considerable loss of crypt, severe damage to the mucosal layer, and a high level of inflammatory cell infiltration. However, supplementation with alginate oligosaccharides prevented intestinal mucosal damage and reduced the histopathological score.

Neutrophil infiltration is the marker of intestinal inflammation in experimental IBD [25]. MPO, as its characteristic enzyme, can induce the production of pro-inflammatory factors and correlates positively with disease severity. It was observed that the expression of MPO in the M group was significantly upregulated, while the treatment with alginate oligosaccharides significantly downgraded the activity of MPO. Furthermore, the balance of the inflammatory factors is also important for maintaining the normal intestinal functions, and imbalance in the regulation of inflammatory factors can lead to the disturbance of the colonic function and destruction of the colonic mucosa, resulting in various clinical manifestations. IL-6 is a pleiotropic regulator of the immune system, acting as both a pro-inflammatory agent by inhibiting T cell apoptosis and as a pro-catabolic and repair agent by acting on the epithelial barrier [26]. The level of IL-1β was found to correlate with disease indices, and high levels of IL-1β may indicate acute injury, while IL-1β gene fragment deletion and blockage of mediated signaling pathways both contribute to the alleviation of DSS-induced colitis symptoms [27]. TNF-α has a pleiotropic effect on intestinal cells: the excess TNF-α changes the integrity of epithelial cells, leading to a weakening of intestinal function [28]. Our results show that three different configurations of alginate oligosaccharides inhibited the secretion of pro-inflammatory cytokines, especially in the GAOS group.

When the intestinal barrier is disrupted and intestinal permeability is increased, LPS will pass through the damaged intestinal mucosa and enter the blood via intestinal epithelial cells to cause damage to the body [29]. Therefore, the expression of LPS can indirectly reflect intestinal permeability. The results of this experiment suggested that the intestinal permeability in the M group increased, while differently structured alginate oligosaccharides could reduce intestinal permeability, and the effect of the GAOS group was more significant. Intestinal mucosal barrier permeability is regulated by tight junctions (TJs), adherent junctions (AJs), and desmosomes [30]. AJs are cell–cell adhesion complexes that participate in regulating cell viability and maintaining tissue integrity [31,32]. E-cadherin is a major component of adhesion junctions (AJs). As a cell-adhesion protein, E-cadherin depletion not only destroys the structure of intestinal epithelial cells but also is associated with impaired function of goblet cells and decreased expression of antimicrobial substances [33]. It has been demonstrated that the reduction in E-cadherin can aggravate the process of UC, which may cause an increase in the intestinal barrier permeability of the colonic epithelium [34,35]. This study indicated that alginate oligosaccharides upgraded the protein expression level of E-cadherin, especially GAOS and HAOS, suggesting that alginate oligosaccharides could improve intestinal permeability by increasing the protein expression level of E-cadherin.

It has been reported that goblet cells protect the host mainly by producing and secreting mucins, after which the mucus layer further isolates micro-organisms from the intestinal epithelium, which serves to protect the intestinal barrier [36,37]. Our results demonstrated that supplementation with alginate oligosaccharides could increase the number of goblet cells. Additionally, the accelerated apoptosis of colonic epithelial cells leads to persistent inflammation in DSS colitis mice. It has been shown that DSS-induced colitis models caused apoptosis of colonic epithelial cells, which in turn disrupted the intestinal mechanical barrier and triggered inflammation of the intestine. The results confirmed that an important therapeutic effect of alginate oligosaccharides on the intestinal barrier is the reduction in epithelium apoptosis via upregulating Bcl-2 and downregulating Bax expression, which is consistent with the results showing that alginate oligosaccharides could reduce intestinal permeability by decreasing the expression of LPS in this study.

SCFAs are mainly produced during the fermentation reaction of bacteria of indigestible dietary fibers such as oligosaccharides, which are important for protecting the intestinal mucosal barrier and maintaining intestinal homeostasis metabolites [38,39]. In addition, SCFAs also affect signal communication among intestinal bacteria [40]. Previous studies have shown that dietary oligosaccharides regulate the production of SCFAs; for example, the content of propionic, butyric, and valeric acids in the cecum of mice with colitis treated with flaxseed oligosaccharides was significantly increased [41]. In our study, alginate oligosaccharides treatment improved the production of SCFAs in the cecum, especially acetate and butyrate. Butyric acid has been reported to be a major source of energy for colonic epithelial cells and can improve intestinal barrier function in DSS-induced colitis by promoting the expression of TJs [42]. The results suggest that alginate oligosaccharides might alleviate intestinal inflammation and promote the repair of the intestinal barrier by reversing the production of SCFAs. Gut microbiota is important for the pathogenesis of UC [43,44,45]. Previous research has reported that the increase in pathogenic bacteria could damage intestinal epithelial cells and accelerate the development of inflammation [46]. The study has illustrated that the abundance and composition of intestinal flora in colitis mice are significantly distinct from that of normal groups [47]. Prebiotics can have a significant therapeutic effect on UC patients by restoring the composition of the intestinal flora. They increase the number of beneficial bacteria, reduce the number of harmful bacteria, and produce SCFAs [48,49]. Our results confirmed this; the β-diversity and species composition at the genus level of intestinal bacteria in the M group of mice differed significantly from the N group. GAOS and HAOS increased the relative abundance of *Romboutsia* and *Lactobacillus*; thus, they prevented the bias of intestinal bacteria. Among them, *Lactobacillus*, as a beneficial bacteria, produces SCFAs and thus has a beneficial effect on the intestinal tract [50]. To further explore whether alginate oligosaccharides administration induces specific microbial changes, LEfSe was used to analyze the relative abundance of key genera. The relative abundance of *Parasutterella*, *Olsenella*, and *Clostridium_sensu_stricto_1* was considered to be positively associated with the severity of colitis [51,52,53,54]. A previous study found that *Clostridium_sensu_stricto_1* was strongly associated with intestinal inflammation and barrier function [55]. The relative abundance of *Clostridium_sensu_stricto_1* was shown to be negatively correlated with the expression level of intestinal tight junction proteins [56]. To our knowledge, the relative abundance of *Parasutterella*, *Clostridium_sensu_stricto_1*, and *Olsenella* was significantly reduced after treatment with GAOS and HAOS. Our study also indicated that the relative abundance of *Ruminoccaceae_NK4A214_group* significantly decreased in the M group, while *Ruminocca-ceae_NK4A214_group* increased following GAOS supplementation. However, the mechanism is unclear. Therefore, more research studies need to explore the mechanism of the *Ruminoccaceae_NK4A214_group*. Our findings indicated that the alleviating effects of alginate oligosaccharides on UC might be achieved by reversing changes in the gut microbiota, especially in some specific microbiota.

## 5. Conclusions

The anti-colitis activities of three types of alginate oligosaccharides against ulcerative colitis induced by 2.5% DSS were investigated in vivo. Our findings suggest that alginate oligosaccharides could significantly relieve UC symptoms in mice. The mechanisms of alginate oligosaccharides might be related to their protection of intestinal epithelial cell integrity, reduction in permeability, reversal of intestinal microbiota changes, and recovery of SCFAs production. These discoveries indicate that alginate oligosaccharides (GSOA, MAOS, and HAOS) may be a potential natural source in functional foods for relieving ulcerative colitis.

## Figures and Tables

**Table 1:** The criteria for disease activity index (DAI) score.

Score	Weight Loss	Fecal State	Bloody Situation
0	<1%	Normal	Negative
1	1–5%	Soft but well-formed	Weak negative
2	5–10%	Soft and shapeless	Positive
3	10–15%	Very loose and moist	Stool bleeding
4	>15%	Diarrhea	Rectal bleeding

**Table 2:** The criteria for histopathology score.

Score	Scoring Criteria
0	Normal
1	Mild inflammation and edema of the mucosal layer; loss of the basal 1/3 of the crypt.
2	Moderate inflammation of the mucosal layer; loss of the basal 2/3 of the crypt.
3	Moderate inflammation of the mucosal layer with complete disappearance of the crypt, but the epithelial layer is still intact.
4	Heavy inflammation of the mucosal, submucosal, and muscular layers; total disappearance of the crypt and epithelium.

## Data Availability

The data of this research are available upon request from the corresponding author.
